# Exploration of a Postbiotic Derived from *Enterococcus faecium* HDRsEf1 and Its Probiotic Mechanisms

**DOI:** 10.3390/microorganisms13071518

**Published:** 2025-06-28

**Authors:** Yingying Chen, Yingting You, Lizhen Ren, Guilin Fu, Naiji Zhou, Yuncai Xiao, Deshi Shi

**Affiliations:** State Key Laboratory of Agriculture Microbiology, College of Veterinary Medicine, Huazhong Agricultural University, Wuhan 430070, China; 1244190308cyy@webmail.hzau.edu.cn (Y.C.); youyingting@webmail.hzau.edu.cn (Y.Y.); rlz34@webmail.hzau.edu.cn (L.R.); fuguilin@webmail.hzau.edu.cn (G.F.); zhounaiji@126.com (N.Z.)

**Keywords:** *Enterococcus faecium* HDRsEf1, exopolysaccharides, intestinal inflammation, cell proliferation, postbiotic, *CXCL-1*

## Abstract

This study aimed to identify the heat-resistant bioactive components of *Enterococcus faecium* HDRsEf1 (HDRsEf1) and investigate their beneficial mechanism. Heat-treated culture supernatants of HDRsEf1 significantly suppressed *CXCL-1* expression in LPS-stimulated MODE-K cells (*p* < 0.001), indicating the presence of heat-resistant anti-inflammatory components. Crude protein (P-Ef1) and crude expolysaccharide (EPS-Ef1) were isolated from an HDRsEf1 culture supernatant using ammonium sulfate and ethanal precipitation. Critically, only crude EPS-Ef1 retained an anti-inflammatory effect after heat treatment, while crude P-Ef1 lost this activity. Further investigation revealed that crude EPS-Ef1 (25 μg/mL) promoted MODE-K cell proliferation via EdU assays (*p* < 0.001), potentially through an upregulation of *PCNA* mRNA expression (*p* < 0.001). Animal studies demonstrated that an oral administration of crude EPS-Ef1 (4 mg/kg bw, 14 days) significantly increased body weight gain and jejunal crypt depth (*p* < 0.05) while reducing intestinal *CXCL-1* mRNA levels (*p* < 0.001). These in vivo findings are consistent with in vitro observations. A structural analysis using HPAEC and SEC-MALLS-RI characterized crude EPS-Ef1 as a heteropolysaccharide (Mw 80.3 kDa) with a near-spherical conformation (slope 0.13) composed of mannose, glucose, glucuronic acid, and galactose (5.4:4.4:1.2:1). In summary, this study identifies crude EPS-Ef1 as the heat-resistant postbiotic component. Crude EPS-Ef1 possesses the dual effects of suppressing intestinal inflammation and promoting intestinal epithelial cell proliferation, which provides a theoretical foundation for a crude EPS-Ef1-based postbiotic.

## 1. Introduction

As the primary interface between the organism and the external environment, the intestinal tract performs a role in nutrient absorption and pathogen defense [1]. In juvenile livestock, developmental immaturity is characterized by impaired tight junctions, deficient mucus secretion, and reduced epithelial turnover [2,3], creating a pathophysiological window that heightens susceptibility to enteric inflammation and pathogenic colonization. Critically, these vulnerabilities result in substantial economic losses due to post-weaning enteritis, which drives the demand for prophylactic interventions. Probiotic supplementation represents a promising strategy [4,5]. As an effective strategy for regulating intestinal microecological balance, probiotic intervention has shown broad application prospects in the field of livestock husbandry in recent years. HDRsEf1, a functional probiotic strain isolated from healthy Tongcheng pig feces, can, mechanistically, effectively reduce the level of intestinal inflammation in piglets by modulating the intestinal flora, reducing potential pathogenic bacteria (e.g., *Escherichia* spp. and *Shigella* spp.) in the piglets’ intestines, and increasing the relative abundance of butyric acid-producing bacteria [6]. Additionally, it also inhibits an LPS-induced down-regulation of ZO-1 expression through the TLR2/4-mediated JNK/AP-1 signaling pathway, thereby protecting the intestinal epithelial barrier [7]. These findings provide an important theoretical basis for the development of microecological preparations based on HDRsEf1.

The application of traditional probiotic products still presents serious challenges. According to the definition of the International Scientific Association for Probiotics and Prebiotics (ISAPP) in 2014, probiotics are “live microorganisms that, when administered in adequate amounts, confer a health benefit on the host” [8]. However, conventional feed processing involving high-temperature pelleting (70–90 °C) induces substantial probiotic inactivation. Research indicates that after 20 min of heat treatment at 70 °C, the number of viable HDRsEf1 decreased by nearly five orders of magnitude. Furthermore, after just 5 min of heat treatment at 80 °C, no viable bacteria were detected. These findings suggest that HDRsEf1 is intolerant to high-temperature environments exceeding 70 °C, which severely limits the commercial application of HDRsEf1 formulations [9]. Notably, the concept of postbiotics provides a new direction to break through this dilemma. The International Scientific Association for Probiotics and Prebiotics (ISAPP) formally defined postbiotics as “preparation of inanimate microorganisms and/or their components that confers a health benefit on the host”, including inanimate microorganisms, bacterial metabolites, and/or components of bacterial lysis in 2021. Studies have confirmed that postbiotics may retain some of the probiotic functions and have better processing stability [10].

The mouse intestinal epithelial cell line (MODE-K) is widely employed to investigate the responses of intestinal epithelial cells to both internal and external stimuli. Lipopolysaccharide (LPS), an endotoxin present in the outer membrane of gram-negative bacteria, has been extensively studied in co-culture with MODE-K cells to activate the NF-κB signaling pathway. This activation subsequently promotes the expression of inflammatory factors, effectively simulating the inflammatory response in the intestine [11,12]. This study established an inflammatory model of intestinal epithelial cells by co-culturing LPS with mouse intestinal epithelial cell lines (MODE-K) in vitro. It screened for heat-resistant components with anti-inflammatory effects derived from HDRsEf1 and further evaluated its intestinal protective effects on juvenile animals through cell proliferation experiments and mouse feeding trials. Finally, high-performance anion-exchange chromatography (HPAEC) and size-exclusion chromatography with multi-angle laser light scattering and refractive index detection (SEC-MALLS-RI) were employed to analyze the compositional and structural features, thereby establishing a foundation for elucidating structure-function relationships.

## 2. Materials and Methods

### 2.1. Cell Line

The mouse small intestinal epithelial cell line (MODE-K) was obtained from Shanghai Hongshun Biotechnology Co., Ltd. (Shanghai, China).

### 2.2. Reagents

MRS broth (Qingdao Haibo Biotechnology Co., Ltd., Qingdao, China). Fetal bovine serum (FBS), DMEM/F-12 medium, and trypsin (Thermo Fisher Scientific, Waltham, MA, USA). Mouse CXCL1/KC (IL-8) ELISA Kit (Absin Bioscience, Shanghai, China). Evo M-MLV Reverse Transcription Kit and SYBR Green Pro Taq HS qPCR Mix (Accurate Biotechnology, Changsha, China). Evo M-MLV Reverse Transcription Kit and SYBR Green Pro Taq HS qPCR Mix (Accurate Biotechnology, China). EdU Cell Proliferation Imaging Kit (green fluorescence) (Yakeyan Biotechnology, Wuhan, China).

### 2.3. Bacterial Strain and Culture

*Enterococcus faecium* HDRsEf1, preserved by the Laboratory of Veterinary Microbiology and Immunology at Huazhong Agricultural University, was activated in MRS broth at 37 °C for 18 h. After two successive subcultures, mid-log-phase bacterial cells were harvested by centrifugation (8000 rpm/min, 4 °C, 5 min), washed twice with sterile PBS, and resuspended in antibiotic-free DMEM medium to a final concentration of 5 × 10^7^ CFU/mL.

### 2.4. Cell Culture

Cryopreserved MODE-K cells were rapidly thawed in a 37 °C water bath, and centrifuged (1000 rpm/min, 5 min). The pellet was resuspended in DMEM/F-12 medium supplemented with 10% FBS and 1% penicillin-streptomycin, then cultured at 37 °C under 5% CO_2_. Cells were passaged at 80% confluence using 0.25% trypsin.

### 2.5. Preparation of Postbiotic Components

#### 2.5.1. Preparation of Crude EPS-Ef1

The ethanol precipitation method was employed to extract extracellular polysaccharides from HDRsEf1 [13]. The activated HDRsEf1 strain was inoculated into fresh MRS liquid medium and cultured overnight in a shaking incubator at 37 °C. The fermentation broth was boiled in a water bath for 10 min to inactivate polysaccharide-degrading enzymes, cooled to room temperature, and centrifuged (10,000 rpm/min, 20 min, 4 °C) to collect the supernatant. Absolute ethanol (3–4 times the volume of the supernatant) was added to the supernatant and left overnight. The mixture was then centrifuged (10,000 rpm/min, 20 min, 4 °C) to collect the precipitate. The precipitate was redissolved, transferred into a dialysis bag (molecular weight cutoff: 3.5 kDa), and dialyzed for 3 days. The dialyzed solution was concentrated and lyophilized under vacuum for storage. Before use, crude EPS-Ef1 was diluted with antibiotic-free DMEM medium, and its concentration was determined using the phenol-sulfuric acid method.

#### 2.5.2. Preparation of Proteins

The ammonium sulfate precipitation method was utilized for the extraction of proteins from the culture supernatant of HDRsEf1 [14]. The activated HDRsEf1 strain was inoculated into fresh MRS liquid medium and cultured overnight in a shaking incubator at 37 °C. The fermentation broth was centrifuged (10,000 rpm/min, 20 min, 4 °C) to remove bacterial cells. The supernatant was stirred on a temperature-controlled magnetic stirrer for 1 h while ammonium sulfate was slowly added. After complete dissolution, the mixture was stored at 4 °C for 4 h. The solution was then centrifuged (10,000 rpm/min, 20 min, 4 °C), and the supernatant was discarded. The precipitate was redissolved, transferred into a dialysis bag (molecular weight cutoff: 3.5 kDa), and dialyzed for 3 days. The dialyzed solution was concentrated and lyophilized under vacuum for storage. Before use, crude P-Ef1 was diluted with antibiotic-free DMEM medium, and its concentration was determined using a BCA protein assay kit (Beyotime Biotechnology, Shanghai, China).

### 2.6. Induction of Inflammatory Cytokines in MODE-K Cells by LPS at Different Concentrations

MODE-K cells were seeded into 12-well tissue culture plates at a density of 1 × 10^5^ cells/mL (1 mL/well) and cultured at 37 °C in a 5% CO_2_ incubator until a confluent monolayer formed. Cells were co-incubated with LPS at concentrations of 0.1 μg/mL, 1 μg/mL, and 10 μg/mL for 2 h. Each treatment was performed in triplicate.

### 2.7. Effects of Heat-Inactivated HDRsEf1 and Its Supernatant on MODE-K Cells 

The cell experiments were designed to include an untreated control group, an LPS-treated group, a group that received live Enterococcus faecium HDRsEf1 (L-Ef1) pretreatment followed by LPS treatment, and groups that received either heat-inactivated Enterococcus faecium HDRsEf1 (D-Ef1) or its supernatant pretreatment followed by LPS treatment.Supernatants from HDRsEf1 cultures at different time points were mixed with DMEM complete medium at a ratio of 1:2 (*v*/*v*). MODE-K cells were first treated withD-Ef1 (5 × 10^7^ CFU/mL) or its supernatant for 2 h. After removal of the culture medium, cells were washed three times with PBS and stimulated with 0.1 μg/mL LPS for 2 h. Triplicates were included for each treatment. The control group for D-Ef1 treatment used DMEM complete medium, while the control for supernatant treatments used a mixture of sterile MRS medium and DMEM complete medium (1:2, *v*/*v*).

### 2.8. Effects of Crude Heat-Inactivated Proteins (HP-Ef1) and Crude EPS-Ef1 on MODE-K Cells

The cell experiments were designed to include an untreated control group, an LPS-treated group, groups that received crude HP-Ef1 pretreatment followed by LPS treatment, and groups that received crude EPS-Ef1 pretreatment followed by LPS treatment. MODE-K cells were treated with crude HP-Ef1 or crude EPS-Ef1 for 2 h. After washing three times with PBS, cells were stimulated with 0.1 μg/mL LPS for 2 h. Triplicates were performed for each treatment, with DMEM complete medium serving as the control.

### 2.9. EdU Assay for Evaluating Crude EPS-Ef1 Effects on MODE-K Cell Proliferation

The 5-ethynyl-2′-deoxyuridine (EdU) assay is a method used to detect proliferating cells by utilizing the incorporation of a thymidine analog, EdU, into newly synthesized DNA during the S-phase of the cell cycle. The alkyne group of EdU participates in a copper-catalyzed ‘click’ reaction with fluorescent azide dyes, allowing for rapid and specific covalent labeling without the need for DNA denaturation [15].

The cell experiments were designed to include an untreated control group and a group that received crude EPS-Ef1 treatment. MODE-K cells were seeded into 12-well plates at 1 × 10^5^ cells/mL (1 mL/well) and cultured until reaching 70–80% confluence. Cells were treated with 25 μg/mL EPS for 2 h. The EdU cell proliferation assay was conducted according to the manufacturer’s instructions, and fluorescence microscopy was employed for detection.

### 2.10. Single-Factor Optimization of Crude EPS-Ef1 Production Conditions

To optimize the culture conditions for the crude EPS-Ef1, single factor tests were performed to investigate the effects of the culture time (12, 24, 36, and 48 h), culture temperature (32, 37, and 42 °C), and seed size (1, 2, 3, and 4%) on the crude EPS-Ef1 yield.

### 2.11. Animal Husbandry and Experimental Design

Twenty-week-old BALB/c mice with similar body weights were randomized using computer-generated sequences and assigned to two groups: a control group (*n* = 10) and a crudeEPS-Ef1-fed group (*n* = 10). Allocation concealment was maintained through coded cage numbering. Blinding procedures involved an independent researcher who prepared and coded all gavage solutions (PBS or 4 mg/kg bw crudeEPS-Ef1 in PBS) with labels A/B. Personnel conducting oral administrations, body weight measurements (every 48 h), and health monitoring were kept unaware of group assignments. Additionally, technicians performing endpoint sample collection and histological analyses were blinded to treatment codes. Group identities were revealed only after the complete data analysis. The mice were housed at a temperature of 23 ± 2 °C under a 12 h light/dark cycle and had ad libitum access to chow and water for 14 days prior to euthanasia on day 15.2.9. Total RNA Extraction and RT-qPCR.

Total RNA was isolated from the MODE-K cells and mouse jejunal tissues using TRIpure reagent, followed by chloroform-isopropanol purification. The RNA quality and concentration were assessed spectrophotometrically. Reverse transcription was performed using the Evo M-MLV RT-Mix Kit. cDNA was subjected to quantitative PCR (qPCR) with SYBR^®^ Green Premix Pro Taq HS qPCR Kit on a real-time PCR system. Primer sequences for reference (*β-actin*) and target genes (*CXCL-1*, *IL-6*, *PCNA*, *Ki67*) are listed in Table 1 (see Table 1 for primer details).

### 2.12. Enzyme-Linked Immunosorbent Assay (ELISA)

Cell culture supernatants were collected into sterile 1.5 mL centrifuge tubes. The expression level of CXCL-1 in supernatants was quantified using a commercial mouse CXCL-1 ELISA kit according to the manufacturer’s instructions.

### 2.13. Hematoxylin and Eosin (H&E) Staining

Jejunal tissues fixed in 4% paraformaldehyde were dehydrated, paraffin-embedded, and sectioned (4 μm thickness). Sections were routinely stained with hematoxylin and eosin (H&E) for histopathological evaluation. Images were captured under a light microscope, and the villus height-to-crypt depth ratio was analyzed using ImageJ software version 1.54f.

### 2.14. Compositional Analysis of Crude EPS-Ef1

EPS samples (1 mg) were dissolved in 1 mL distilled water. Monosaccharide composition was analyzed using a Thermo ICS5000 ion chromatography system (Thermo Fisher Scientific, USA) equipped with a Dionex™ CarboPac™ PA20 column (150 × 3.0 mm, 10 μm). Standards were included for peak identification and quantification [16].

### 2.15. Characterization of Molecular Weight and Conformation of Crude EPS-Ef1

The molecular weight distribution of crude EPS-Ef1 was analyzed using a gel permeation chromatography system coupled with multi-angle laser light scattering (GPC-MALLS; U3000, Thermo Fisher Scientific, USA). A refractive index (RI) detector measured concentration, while MALLS provided light scattering data. Molecular weights were calculated using the Mark–Houwink equation. Calibration curves were generated using standard dextran solutions (concentration vs. peak area) for quantitative analysis [16].

### 2.16. Statistical Analysis

Data analysis was performed using SPSS version 26.0. Outliers were first identified using box plots (box-and-whisker plots), with data points exceeding 1.5 times the interquartile range (IQR) above the third quartile or below the first quartile marked as potential outliers. After validating their validity, these outliers were either excluded or retained. The Shapiro–Wilk test was then used to assess the normality of the data across groups. Unless otherwise noted, research results are presented as mean ± SD. For comparisons between two groups, Student’s *t*-test was used for normally distributed data, while non-parametric tests were applied for non-normally distributed data. For multiple comparisons, one-way analysis of variance (ANOVA) with Bonferroni post-hoc test was employed to assess significance. Statistical significance is denoted by asterisks: * *p* < 0.05, ** *p* < 0.01, and *** *p* < 0.001.

## 3. Results

### 3.1. Cell Experiments

#### 3.1.1. LPS Upregulates CXCL-1 mRNA Levels in MODE-K Cells

**Figure 1:**
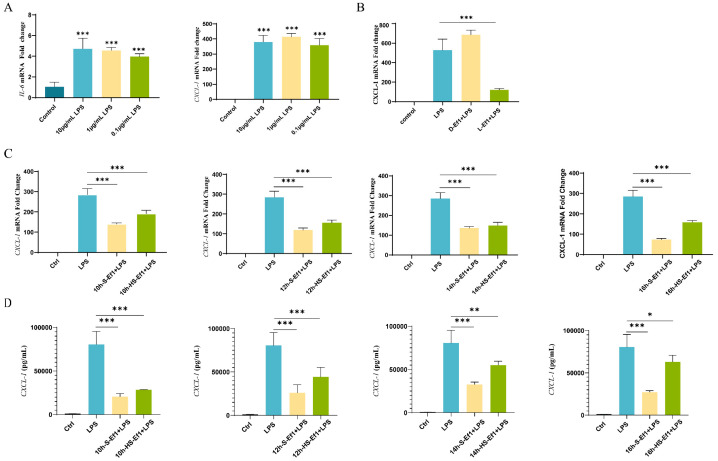
HS-Ef1 retain the ability to suppress *CXCL-1* overexpression in MODE-K cells. (**A**) Relative mRNA expression levels of *IL-6* and *CXCL-1* in MODE-K cells treated with varying LPS concentrations (0.1, 1, and 10 μg/mL) for 2 h. (**B**) Relative *CXCL-1* mRNA expression in MODE-K cells pre-treated with L-Ef1 or D-Ef1 for 2 h, followed by 0.1 μg/mL LPS stimulation for 2 h. (**C**) Relative *CXCL-1* mRNA expression and (**D**) protein levels in MODE-K cells pre-treated with S-Ef1 or HS-Ef1 collected at different culture times (10, 12, 14, and 16 h), followed by 0.1 μg/mL LPS stimulation for 2 h. * *p* < 0.05, ** *p* < 0.01, and *** *p* < 0.001.

Lipopolysaccharide (LPS), a classic pathogen-associated molecular pattern (PAMP) released by gram-negative bacteria, specifically binds to Toll-like receptor 4 (TLR4) in the host immune system, activating TLR4-mediated NF-κB and MAPK signaling pathways. This activation induces the production of inflammatory cytokines and chemokines, thereby exacerbating inflammatory responses [17,18]. To determine the minimum LPS concentration required to induce inflammatory responses in MODE-K cells, the cells were treated with varying LPS concentrations (0.1 μg/mL, 1 μg/mL, 10 μg/mL) for 2 h. An RT-qPCR analysis revealed that LPS exposure significantly upregulated the mRNA expression levels of *IL-6* and *CXCL-1*. Notably, *CXCL-1* exhibited remarkable sensitivity: its mRNA expression increased 300–400-fold (according to ΔΔCt values) in response to the lowest LPS concentration (0.1 μg/mL) compared to the untreated group (Figure 1A). Based on these findings, 0.1 μg/mL LPS was selected for subsequent experiments, with *CXCL-1* expression designated as the core indicator of the inflammatory status of intestinal epithelial cells.

#### 3.1.2. D-Ef1 Fails to Suppress CXCL-1 Overexpression in MODE-K Cells

Previous studies demonstrated that live L-Ef1 alleviates inflammation in porcine intestinal epithelial cells (IPEC-J2) [19]. Here, we observed a similar anti-inflammatory effect of HDRsEf1 in MODE-K cells. To evaluate whether D-Ef1 retains this activity, MODE-K cells were pre-treated with D-Ef1. The results indicate that the mRNA expression level of *CXCL-1* in the LPS-treated group increased by 527-fold compared to the control group. In the heat-inactivated HDRsEf1 pre-treatment group, the expression level of *CXCL-1* at the mRNA level rose by 686-fold relative to the control group. Notably, there was no significant difference in the *CXCL-1* expression levels between the LPS-treated group and the heat-inactivated HDRsEf1 pre-treatment group. Therefore, it can be concluded that a pre-treatment of MODE-K cells with heat-inactivated HDRsEf1 does not inhibit the LPS-induced overexpression of *CXCL-1* (Figure 1B).

#### 3.1.3. HDRsEf1-Derived Culture Supernatants Inhibit CXCL-1 Expression in MODE-K Cells

To investigate whether HDRsEf1-derived supernatants (HS-Ef1) retain an anti-inflammatory capacity, MODE-K cells were pre-treated with supernatants collected at different culture times (10 h, 12 h, 14 h, and 16 h of shaking incubation). Both HS-Ef1 and non-heat-inactivated HDRsEf1-derived supernatants (S-Ef1) significantly suppressed LPS-induced *CXCL-1* overexpression at mRNA and protein levels. It is noteworthy that the inhibitory effect of HS-Ef1 is less pronounced than that of S-Ef1. For instance, in MODE-K cells pretreated with S-Ef1 and harvested after 10 h of shaking culture, the mRNA expression level of *CXCL-1* was reduced by 50.88% compared to the LPS-treated group, while the protein expression level exhibited a reduction of 74.48% relative to the LPS-treated group. In contrast, MODE-K cells treated with HS-Ef1 and collected after the same duration of shaking culture showed only a 33.33% reduction in mRNA expression of *CXCL-1* compared to the LPS-treated group, and the protein expression level was reduced by 64.71% in comparison to the LPS-treated group. Similar experimental results were observed when MODE-K cells were pretreated with HDRsEf1 culture supernatant collected at different culture times. (Figure 1C,D).

#### 3.1.4. Crude HP-Ef1 Fail to Suppress CXCL-1 Overexpression in MODE-K Cells

**Figure 2:**
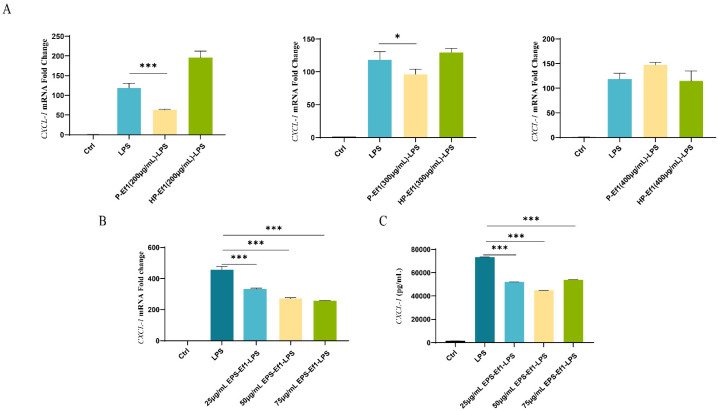
Effects of crude EPS-Ef1 and crude HP-Ef1 on CXCL-1 secretion in MODE-K cells. (**A**) Relative mRNA expression of *CXCL-1* in MODE-K cells pre-treated with varying concentrations of crude P-Ef1 or crude HP-Ef1 (200, 300, and 400 μg/mL) for 2 h, followed by 0.1 μg/mL LPS stimulation for 2 h. (**B**) Relative *CXCL-1* mRNA expression. (**C**) Protein levels in MODE-K cells pre-treated with crudeEPS-Ef1 at different concentrations (25, 50, and 75 μg/mL) for 2 h, followed by 0.1 μg/mL LPS stimulation for 2 h. * *p* < 0.05, and *** *p* < 0.001.

To identify key bioactive components in HDRsEf1-derived supernatants, secretory proteins were isolated via ammonium sulfate precipitation. At concentrations of 200 μg/mL and 300 μg/mL, crudeP-Ef1 significantly inhibited LPS-induced *CXCL-1* overexpression at both mRNA and protein levels. However, crude HP-Ef1 lost this inhibitory activity (Figure 2A).

#### 3.1.5. Crude EPS-Ef1 Suppress CXCL-1 Overexpression in MODE-K Cells

Previous studies indicate that EPS released by certain probiotics can directly or indirectly modulate intestinal inflammatory responses in humans and animals [20]. In this study, the pre-treatment of MODE-K cells with crude EPS-Ef1 at varying concentrations (25, 50, and 75 μg/mL) significantly attenuated the LPS-induced overexpression of *CXCL-1* at both mRNA and protein levels, as demonstrated by RT-qPCR and ELISA (Figure 2B,C). Notably, the inhibitory effect exhibited a dose-dependent trend. The highest inhibition efficiency was achieved with 75 μg/mL crudeEPS-Ef1 pretreatment, reaching 43.76% (Figure 2B).

### 3.2. Crude EPS-Ef1 Enhances MODE-K Cell Proliferation

#### 3.2.1. EdU Assay for Cell Proliferation

**Figure 3:**
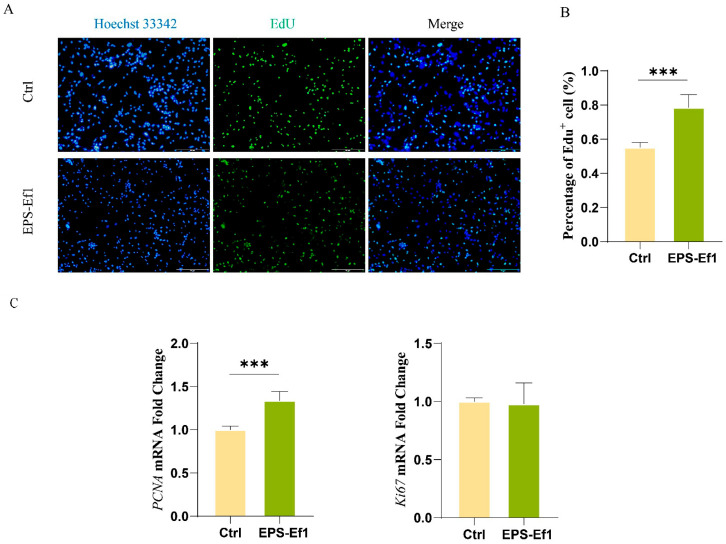
Crude EPS-Ef1 promotes MODE-K cell proliferation (*n* = 5). (**A**) Proliferation of MODE-K cells assessed by EdU assay. (**B**) Proportion of proliferating cells quantified using Image J. (**C**) Relative mRNA expression of *PCNA* and *Ki67* in MODE-K cells (*n* = 3). *** *p* < 0.001.

The proliferation of MODE-K cells treated with 25 μg/mL crude EPS-Ef1 was assessed using the EdU assay. Blue indicates Hoechst 33342-stained cell nuclei, while red denotes proliferating cells. Treatment with 25 μg/mL EPS-Ef1 significantly increased the EdU^+^ cell rate from 55.28% in the control group to 78.55%, demonstrating a statistically significant difference (*p* < 0.001) (Figure 3A).

#### 3.2.2. Crude EPS-Ef1 Upregulates PCNA mRNA Expression

Compared to the control group, treatment with 25 μg/mL EPS-Ef1 significantly increased the expression level of *PCNA* at the mRNA level (*p* < 0.001). However, there was no significant effect on *Ki67* expression at the mRNA level. This discrepancy may be attributed to the differing kinetics of these two proliferation markers. Specifically, *PCNA* demonstrates a rapid response to the S phase process within a few hours, whereas *Ki67* may necessitate prolonged stimulation to accurately indicate entry into the cell cycle [21] (Figure 3B). These results confirm that crude EPS-Ef1 promotes MODE-K cell proliferation.

### 3.3. Optimization of Crude EPS-Ef1 Yield Through Cultivation Condition Modifications

**Figure 4:**
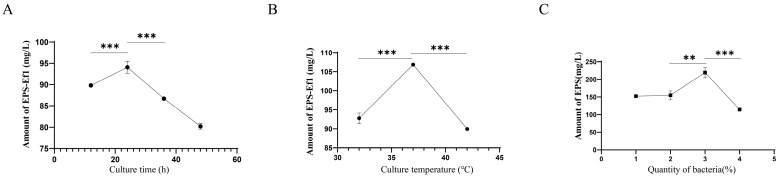
Single-factor optimization of crude EPS-Ef1 production conditions (*n* = 3). (**A**) Crude EPS-Ef1 yield under different culture time (12, 24, 36, and 48 h). (**B**) Crude EPS-Ef1 yield at varying cultivation temperatures (32, 37, and 42 h). (**C**) Crude EPS-Ef1 yield with different inoculum sizes (1, 2, 3, and 4%). ** *p* < 0.01, and *** *p* < 0.001.

Extensive studies demonstrate that the culture time significantly influences the EPS yield. During the lag and early logarithmic growth phases, bacterial metabolism prioritizes the synthesis of essential proteins for proliferation, resulting in minimal EPS production. Conversely, EPS synthesis accelerates during the late logarithmic and stationary phases but declines during the death phase due to reduced synthesis and enhanced degradation. Single-factor experiments were conducted to determine the optimal culture time [22]. As shown in Figure 4A, crude EPS-Ef1 production peaked at 24 h (94.07 mg/L), which was subsequently selected for further studies.

The environmental temperature critically impacts microbial EPS synthesis. Lower temperatures reduce metabolic activity, slowing EPS production, while excessively high temperatures disrupt cellular integrity. Strain-specific temperature adaptability necessitates optimization. Our results revealed maximal crudeEPS-Ef1 yield (106.86 mg/L) at 37 °C (Figure 4B), establishing this as the optimal temperature.

Inoculum size also modulates EPS synthesis. Adequate nutrients and space support bacterial growth and EPS production, whereas excessive inoculum induces nutrient competition and accelerated depletion, suppressing yield. As demonstrated in Figure 4C, a 3% inoculum of HDRsEf1 achieved the highest crude EPS-Ef1 production (219.55 mg/L). In summary, under optimized conditions (24 h, 37 °C, 3% inoculum), crude EPS-Ef1 production reached 219.55 mg/L, representing the highest yield in this study.

### 3.4. Animal Experiments

#### 3.4.1. Crude EPS-Ef1 Promotes Body Weight Gain in Mice

**Figure 5:**
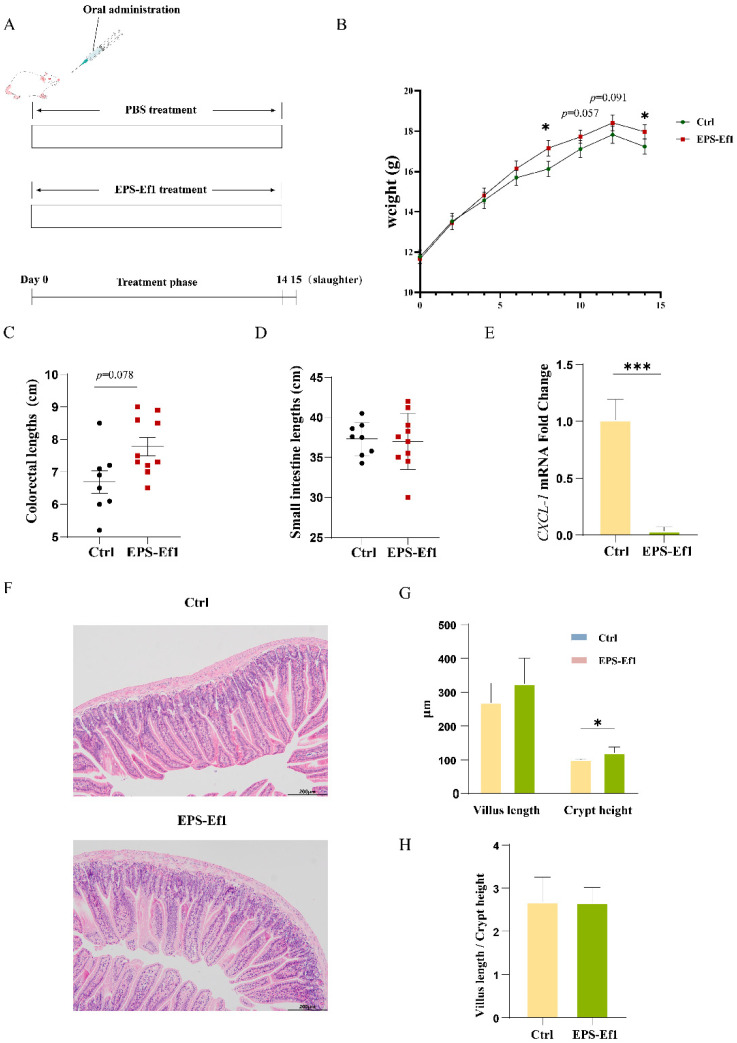
Daily intake of 4 mg/kg bw crudeEPS-Ef1 suppresses intestinal *CXCL-1* expression and enhances epithelial cell proliferation in mice. (**A**) Schematic of the experimental timeline for BALB/c mice. (**B**) Body weight changes in BALB/c mice recorded at 0, 2, 4, 6, 8, 10, 12, and 14 days post-treatment. (**C**) Colon length and (**D**) small intestinal length measurements. (**E**) RT-qPCR analysis of *CXCL-1* mRNA levels in jejunal tissue (*n* ≥ 4). (**F**–**H**) Representative H&E-stained jejunal sections from crudeEPS-Ef1-treated mice, depicting changes in villus length, crypt depth, and villus-to-crypt ratio (scale bar = 200 μm; *n* = 5). Data are presented as mean ± SEM. * *p* < 0.05, and *** *p* < 0.001.

No significant difference in initial body weight was observed between experimental and control groups. However, crude EPS-Ef1-treated mice exhibited significantly higher body weights at days 8 and 14 (*p* < 0.05), with a consistent upward trend at days 10 and 12 (Figure 5B).

#### 3.4.2. Crude EPS-Ef1 Enhances Intestinal Crypt Depth in Mice

Crude EPS-Ef1 treatment increased colorectal length (Figure 5C) but did not significantly alter small intestinal length (Figure 5D). Histological analysis (H&E staining) revealed significantly deeper crypts in the small intestine (*p* < 0.05), alongside trends toward increased villus length (Figure 5F–H). It is hypothesized that the observed increase in villus length may have contributed to an enhancement of the intestinal epithelial area, thereby facilitating improved nutrient absorption.

#### 3.4.3. Crude EPS-Ef1 Reduces CXCL-1 Expression in Mouse Jejunum

RT-qPCR confirmed that crude EPS-Ef1 significantly suppressed *CXCL-1* mRNA levels in the jejunum (*p* < 0.001), consistent with in vitro findings (Figure 5E).

### 3.5. Analysis of Monosaccharide Composition and Structure of Crude EPS-Ef1

#### 3.5.1. Monosaccharide Composition Analysis

**Figure 6:**
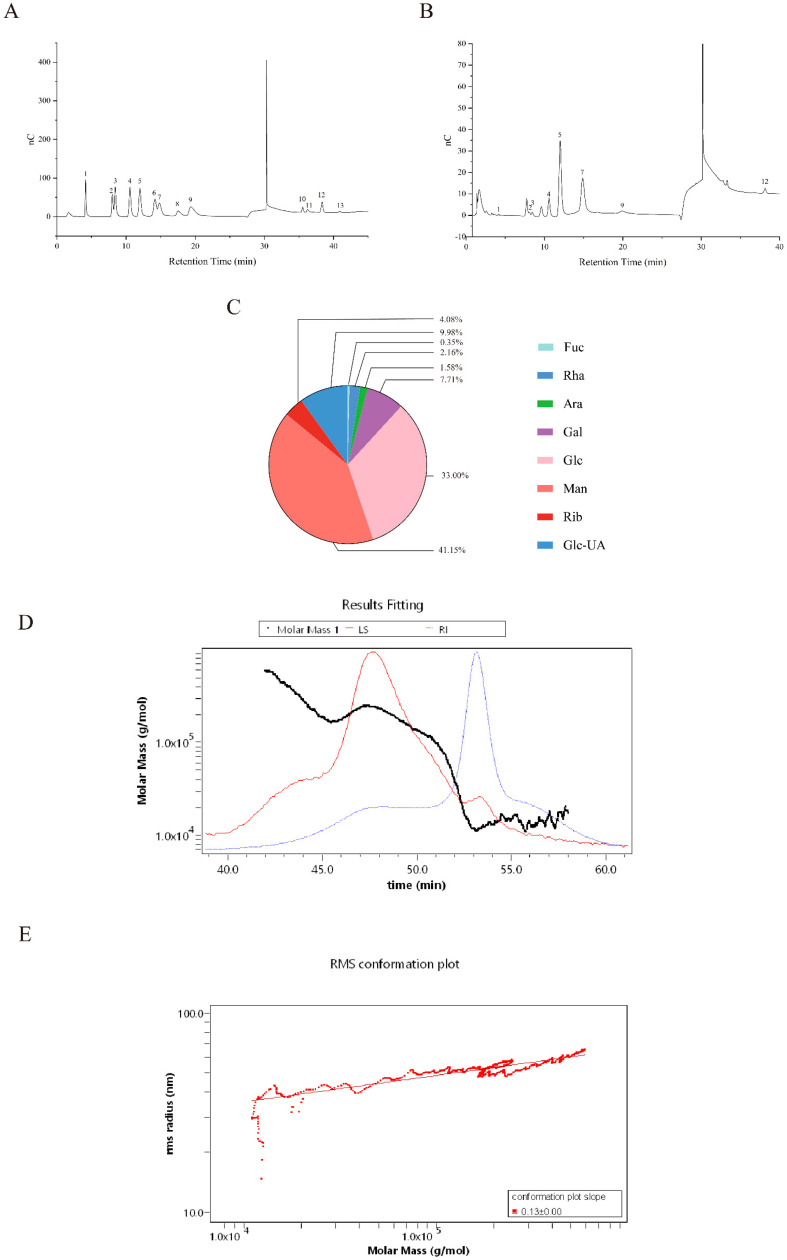
Analysis of monosaccharide composition, molecular weight, and molecular conformation of crude EPS-Ef1. (**A**) High-performance anion-exchange chromatography (HPAEC) profile of monosaccharide standards. (**B**) HPAEC chromatogram of crude EPS-Ef1. (**C**) Monosaccharide composition and relative abundance in crude EPS-Ef1. Peaks 1–12 correspond to the following monosaccharides: 1, fucose (Fuc); 2, rhamnose (Rha); 3, arabinose (Ara); 4, galactose (Gal); 5, glucose (Glc); 6, xylose (Xyl); 7, mannose (Man); 8, fructose (Fru); 9, ribose (Rib); 10, guluronic acid (GulA); 11, glucuronic acid (GlcA); 12, galacturonic acid/mannuronic acid (GalA/ManA). (**D**) Molar mass distribution profile of crude EPS-Ef1 determined by size-exclusion chromatography. (**E**) Molecular conformation analysis of crude EPS-Ef1 based on a log–log plot of molar mass versus root mean square (RMS) radius.

Crude EPS-Ef1 was hydrolyzed with trifluoroacetic acid and analyzed via high-performance anion-exchange chromatography (HPAEC). Peaks in the chromatogram (Figure 6B) corresponded to fucose (Fuc), rhamnose (Rha), arabinose (Ara), galactose (Gal), glucose (Glc), mannose (Man), ribose (Rib), and galacturonic acid (Glc-UA) derivatives (Figure 6A). Quantification of 547.73 μg crude EPS-Ef1 revealed the following composition: Man (41.15%), Glc (33.00%), Gal (7.71%), Glc-UA (9.98%), Rib (4.08%), Rha (2.16%), Ara (1.58%), and Fuc (0.35%) (Figure 6C). Molar ratios were determined as Man:Glc:Glc-UA:Gal = 5.43:4.34:1.22:1, highlighting its predominant monosaccharide profile.

#### 3.5.2. Molecular Structural Characteristics of Crude EPS-Ef1

Crude EPS-Ef1 exhibited a weight-average molecular weight (Mw) of 80.305 kDa and a number-average molecular weight (Mn) of 21.81 kDa. The calculated polydispersity index (Mw/Mn ratio) of 3.682 indicates that crude EPS-Ef1 exhibits a broad molecular weight distribution, which is characteristic of heterogeneous polymer. (Figure 6D). Analysis of molecular conformation via log–log plots of molar mass versus hydrodynamic radius revealed a slope of 0.13, suggesting a near-spherical molecular conformation (Figure 6E).

## 4. Discussion

With the proposed concept of postbiotics, investigations focused on inactivated probiotics and their metabolites have emerged as a research priority in microbial sciences. As a candidate for a new generation of microecological agents, the thermal stability characteristics of postbiotics offer a critical solution for the industrialized production of feed additives. Therefore, the screening of thermally tolerant postbiotics from the HDRsEf1 strain may address the technical barrier of processing-induced functional deactivation.

In this study, we found that *CXCL-1* mRNA expression in MODE-K cells showed a significant 300–400-fold (according to ΔΔCt values) upregulation in comparison with the control group after stimulation by LPS, suggesting that CXCL-1 can be used as a susceptibility indicator to assess intestinal inflammation. Mechanistically, CXCL-1 binds CXCR2 to mediate neutrophil chemotaxis during inflammation. Moderate neutrophil infiltration contributes to pathogen clearance, but excessive aggregation exacerbates tissue damage by releasing mediators such as matrix metalloproteinases and reactive oxygen species [20,23]. Therefore, a precise regulation of *CXCL-1* expression is important for maintaining intestinal homeostasis. Thermal tolerance screening showed that while heat-treated HDRsEf1 and crude P-Ef1 lost anti-inflammatory activity, high-temperature-extracted crude EPS-Ef1 maintained a significant, dose-dependent inhibition of *CXCL-1* expression at both mRNA and protein levels (*p* < 0.001). Given that *CXCL-1* is a downstream effector molecule of the NF-κB signaling pathway, it is hypothesized that crude EPS-Ef1 may attenuate the expression of *CXCL-1* by inhibiting the activity of the NF-κB signaling pathway [24]. This finding corresponds to previous studies that EPS is a key effector molecule for probiotics in mediating beneficial effects. For example, the EPS of *Lactobacillus plantarum* N14 can inhibit *IL-6* and *IL-8* production by upregulating TLR negative regulators [25], and the EPS of *Bacillus albus* DM-15 has both antioxidant and antitumor activities [26]. In this study, we further found that crude EPS-Ef1 upregulates *PCNA* mRNA, a marker of S-phase progression, suggesting enhanced DNA replication in MODE-K cells. This functional outcome is characterized by stimulated proliferation, paralleling reports that EPS-A28 derived from *Alteromonas* induces fibroblast proliferation by enhancing *Ki67* expression. Notably, despite their differing monosaccharide compositions—EPS-A28 is a heteropolysaccharide composed of mannuronic acid, glucose, and N-acetylglucosamine in a molar ratio of 1:3.67:0.93, whereas crude EPS-Ef1 is composed of mannose, glucose, glucuronic acid, and galactose in a molar ratio of 5.43:4.43:1.22:1—both polysaccharides exhibit proliferative bioactivity. This suggests that specific structural motifs, such as the glucan backbone, may universally participate in proliferative signaling pathways. Future studies should elucidate whether conserved domains are responsible for these effects across different cell types [27].

Although existing studies have demonstrated that EPS from probiotics have significant beneficial effects, their industrial application in livestock and poultry farming is still limited by low yield and complex purification processes. The literature data show that the EPS yields of common probiotics usually range from 50–1000 mg/L. For example, the EPS yields of several strains of *Bifidobacterium bifidum* isolated from infant feces and breast milk by Gulcin Alp et al. only ranged from 38.00–97.64 mg/L [28]. And *Lactobacillus plantarum* NTMI05 produced only 895 mg/L of EPS in optimized medium [29]. The production of EPS from *Enterococcus faecium* WEFA23 isolated from infant feces was also only at 130 mg/L [30]. In this study, by single-factor optimization of conditions such as cultivation time, cultivation temperature, and the amount of inoculum of HDRsEf1, we succeeded in increasing the yield of crude EPS-Ef1 from 90.37 mg/L to 219.55 mg/L, which was at the average level of the EPS yield of probiotics. Significantly, the in vivo experimental data revealed that crude EPS-Ef1 exerted substantial beneficial effects at an administered dose of 4 mg/kg bw. It induced a significant increase in the depth of jejunal crypts in mice without affecting the ratio of intestinal villus length and crypt depth (*p* < 0.05). Considering that the crypt is the most proliferative area of intestinal epithelial cells [31], this morphological alteration suggests that crudeEPS-Ef1 may expand the area of nutrient absorption by enhancing the proliferative capacity of jejunal epithelial cells, thereby enhancing the body weight gain (*p* < 0.05). Meanwhile, the down-regulation of *CXCL-1* mRNA expression level in jejunal tissues further validated the anti-inflammatory effect of crude EPS-Ef1 in vivo. In particular, it should be noted that the effective dose of crude EPS-Ef1 in vivo (4 mg/kg bw) in this study was much lower than that of EPS from other microorganisms, suggesting that it has a significant advantage in industrialized applications. Comparative research has shown that *Lactobacillus plantarum* JLAU103-derived EPS requires a dose of 300 mg/kg bw-600 mg/kg bw to ameliorate glycolipid metabolism disorders and neurological damage in diabetic mice [32]. An administration of 150 mg/kg bw birch-derived EPS (IOP) was required to inhibit the expression of inflammatory mediators in the colonic tissues of a mouse model of colitis-associated cancer (CAC) [33]. These dose-response disparities indicate that crude EPS-Ef1 exhibits a superior dose-economic profile while retaining a significant beneficial effect, which provides an important theoretical foundation for its large-scale industrial application in livestock and poultry farming.

Research has shown that the functions of bioactive components are closely related to their specific structural compositions, and the structural analysis of crude EPS-Ef1 is conducive to the understanding of its structure–effect relationships. An analysis using HPAEC and SEC-MALLS-RI demonstrated that crude EPS-Ef1 is a heterogeneous polysaccharide composed of mannose, glucose, glucuronic acid, and galactose in a molar ratio of 5.43:4.43:1.22:1, with a molecular weight (Mw) of 80.305 kDa and a near-spherical conformation. The monosaccharide composition analysis indicated that crude EPS-Ef1 predominantly consisted of mannose (44.95%) and glucose (36.67%). Notably, its structural composition resembled that of the established mannan oligosaccharide (MOS), offering mechanistic insights into the observed biological functions. It has been shown that MOS promotes macrophage polarization to the M2 type by targeting the SIGNR1 receptor in a DSS-induced murine colitis model, which in turn down-regulates the expression of pro-inflammatory factors such as TNF-α and IL-6 [34]. In addition, a deepening of intestinal crypts was observed in chicks after MOS supplementation [35]. These findings are congruent with the effect of crude EPS-Ef1 in inhibiting the expression of the intestinal pro-inflammatory factor CXCL-1 and promoting the proliferation of intestinal epithelial cells in mice. Further analysis revealed that the presence of a minor proportion of glucuronic acid residues (10.12%) may potentially mediate the anti-inflammatory and antioxidant functions of crude EPS-Ef1 by enhancing the molecular negativity promoting its electrostatic binding to positively charged inflammatory mediators [36]. An analysis of its molecular weight and conformation revealed that the low molecular weight (80.305 kDa) and near-spherical conformation of crude EPS-Ef1 may also be the structural foundation for its beneficial effects. This compact 3D structure effectively mitigates steric hindrance effects commonly observed in high molecular weight polysaccharides (≥100 kDa), thereby ensuring effective molecular recognition by membrane receptors [37,38,39].

Despite the remarkable functional properties of crude EPS-Ef1 revealed in this study, the limitations of the current research must be acknowledged. First, although CXCL-1 as a key biomarker reliably reflects the level of intestinal epithelial inflammation, its analysis should be integrated with IL-17 and IL-22 and other inflammatory factors related to intestinal lamina propria lymphocytes through a multifaceted analysis [4,40]. Second, the specific signaling pathway of the pro-proliferative effect of crude EPS-Ef1 remains incompletely elucidated. The synergistic role of the Wnt/β-catenin and Notch pathways in the process deserves further investigation [41,42]. In addition, although crude EPS-Ef1 demonstrates thermostability, its thermostability mechanism has not been fully elucidated. For example, whether the heat resistance of crude EPS-Ef1 is directly related to its molecular weight, monosaccharide composition, or branching structure still needs further investigation [43].

In summary, this study is the first to confirm that crude EPS-Ef1, a thermotolerant heteropolysaccharide, exhibits both anti-inflammatory effects by inhibiting the expression of the pro-inflammatory factor CXCL-1 in vitro and in vivo, as well as pro-proliferative effects by stimulating cell proliferation through increased *PCNA* expression at the mRNA level, even at low doses. The unique monosaccharide composition and molecular conformation of crude EPS-Ef1 may serve as the structural basis for its probiotic effects. Compared with conventional probiotics, the thermostability of crude EPS-Ef1 mitigates thermal degradation during feed pelleting (70–90 °C), while its low-dose efficacy significantly reduces production costs. These attributes hold substantial potential for advancing the commercial scalability of postbiotic preparations in livestock production systems.

## Figures and Tables

**Table 1:** List of genes and primer sequence for quantitative real-time PCR analysis.

Gene Name	Forward Primer (5′–3′)	Reverse Primer (5′–3′)	mRNA ID
*β-actin*	TGAGCTGCGTTTTACACCCT	GCCTTCACCGTTCCAGTTTT	NM_007393.5
*CXCL-1*	ACTGCACCCAAACCGAAGTC	TGGGGACACCTTTTAGCATCTT	NM_008176.3
*IL-6*	GTCGGAGGCTTAATTACACA	TTTTCTGCAAGTGCATCATC	NM_001314054.1
*PCNA*	GCCGAGACCTTAGCCACATT	GTAGGAGACAGTGGAGTGGC	NM_011045.2
*Ki67*	ACCGTGGAGTAGTTTATCTGGG	TGTTTCCAGTCCGCTTACTTCT	NM_026472.4

## Data Availability

The original contributions presented in this study are included in the article. Further inquiries can be directed to the corresponding authors.

## References

[1] Shi N., Li N., Duan X., Niu H. (2017). Interaction between the Gut Microbiome and Mucosal Immune System. Mil. Med. Res..

[2] Li Y., Shi P., Yao K., Lin Q., Wang M., Hou Z., Tang W., Diao H. (2024). Diarrhea Induced by Insufficient Fat Absorption in Weaned Piglets: Causes and Nutrition Regulation. Anim. Nutr..

[3] Wang Q., Wang F., Tang L., Wang Y., Zhou Y., Li X., Jin M., Fu A., Li W. (2024). *Bacillus amyloliquefaciens* SC06 Alleviated Intestinal Damage Induced by Inflammatory via Modulating Intestinal Microbiota and Intestinal Stem Cell Proliferation and Differentiation. Int. Immunopharmacol..

[4] Hou Q., Ye L., Liu H., Huang L., Yang Q., Turner J.R., Yu Q. (2018). *Lactobacillus* Accelerates ISCs Regeneration to Protect the Integrity of Intestinal Mucosa through Activation of STAT3 Signaling Pathway Induced by LPLs Secretion of IL-22. Cell Death Differ..

[5] Wu H., Xie S., Miao J., Li Y., Wang Z., Wang M., Yu Q. (2020). *Lactobacillus reuteri* Maintains Intestinal Epithelial Regeneration and Repairs Damaged Intestinal Mucosa. Gut Microbes.

[6] Zhou J., Luo J., Yang S., Xiao Q., Wang X., Zhou Z., Xiao Y., Shi D. (2021). Different Responses of Microbiota across Intestinal Tract to *Enterococcus faecium* HDRsEf1 and Their Correlation with Inflammation in Weaned Piglets. Microorganisms.

[7] Liu L., Li Y., He Y., Wang Z., Zhao H., Jin X., Shi D., Wang X. (2022). *Enterococcus faecium* HDRsEf1 Inhibits Lipopolysaccharide-Induced Downregulation of Zona Occludens -1 Expression via Toll-like Receptor 2/4-Mediated c-Jun N-Terminal Kinase/Activator Protein-1 Signalling Pathways. J. Appl. Microbiol..

[8] Hill C., Guarner F., Reid G., Gibson G.R., Merenstein D.J., Pot B., Morelli L., Canani R.B., Flint H.J., Salminen S. (2014). Expert Consensus Document. The International Scientific Association for Probiotics and Prebiotics Consensus Statement on the Scope and Appropriate Use of the Term Probiotic. Nat. Rev. Gastroenterol. Hepatol..

[9] Wang T., Xiong Y., Xiao Y., Wang X., Bi D., Shi D. (2017). Probiotic characteristics of *Enterococcus faecium* HDRsEf1 from swine feces. Biot. Resour..

[10] Vinderola G., Sanders M.E., Salminen S. (2022). The Concept of Postbiotics. Foods.

[11] Yang L., Wu G., Wu Q., Peng L., Yuan L. (2022). METTL3 Overexpression Aggravates LPS-Induced Cellular Inflammation in Mouse Intestinal Epithelial Cells and DSS-Induced IBD in Mice. Cell Death Discov..

[12] Miao F., Shan C., Ning D. (2021). Walnut Oil Alleviates LPS-Induced Intestinal Epithelial Cells Injury by Inhibiting TLR4/MyD88/NF-κB Pathway Activation. J. Food Biochem..

[13] Jiang G., Li R., He J., Yang L., Chen J., Xu Z., Zheng B., Yang Y., Xia Z., Tian Y. (2022). Extraction, Structural Analysis, and Biofunctional Properties of Exopolysaccharide from *Lactiplantibacillus pentosus* B8 Isolated from Sichuan Pickle. Foods.

[14] Diao H., Xing P., Tian J., Han Z., Wang D., Xiang H., Liu T., Ma R. (2022). Toxicity of Crude Toxin Protein Produced by *Cordyceps fumosorosea* IF-1106 Against *Myzus persicae* (Sulze). J. Invertebr. Pathol..

[15] Troth E.V., Kyle D.E. (2021). EdU Incorporation to Assess Cell Proliferation and Drug Susceptibility in Naegleria Fowleri. Antimicrob. Agents Chemother..

[16] Du B., Fu Y., Wang X., Jiang H., Lv Q., Du R., Yang Y., Rong R. (2019). Isolation, Purification, Structural Analysis and Biological Activities of Water-Soluble Polysaccharide from *Glehniae radix*. Int. J. Biol. Macromol..

[17] Mao N., Yu Y., Lu X., Yang Y., Liu Z., Wang D. (2024). Preventive Effects of Matrine on LPS-Induced Inflammation in RAW 264.7 Cells and Intestinal Damage in Mice through the TLR4/NF-κB/MAPK Pathway. Int. Immunopharmacol..

[18] Jack R.S., Fan X., Bernheiden M., Rune G., Ehlers M., Weber A., Kirsch G., Mentel R., Fürll B., Freudenberg M. (1997). Lipopolysaccharide-Binding Protein Is Required to Combat a Murine Gram-Negative Bacterial Infection. Nature.

[19] Tian Z., Liu X., Dai R., Xiao Y., Wang X., Bi D., Shi D. (2016). *Enterococcus faecium* HDRsEf1 Protects the Intestinal Epithelium and Attenuates ETEC-Induced IL-8 Secretion in Enterocytes. Mediat. Inflamm..

[20] Li H., Li H., Stanton C., Ross R.P., Zhao J., Chen W., Yang B. (2024). Exopolysaccharides Produced by *Bifidobacterium longum* subsp. *longum* YS108R Ameliorates DSS-Induced Ulcerative Colitis in Mice by Improving the Gut Barrier and Regulating the Gut Microbiota. J. Agric. Food Chem..

[21] Kurki P., Vanderlaan M., Dolbeare F., Gray J., Tan E.M. (1986). Expression of Proliferating Cell Nuclear Antigen (PCNA)/Cyclin during the Cell Cycle. Exp. Cell Res..

[22] Chen L., Gu Q., Zhou T. (2022). Statistical Optimization of Novel Medium to Maximize the Yield of Exopolysaccharide From *Lacticaseibacillus rhamnosus* ZFM216 and Its Immunomodulatory Activity. Front. Nutr..

[23] Zhu Y., Yang S., Zhao N., Liu C., Zhang F., Guo Y., Liu H. (2021). CXCL8 Chemokine in Ulcerative Colitis. Biomed. Pharmacother..

[24] Kemp S.B., Carpenter E.S., Steele N.G., Donahue K.L., Nwosu Z.C., Pacheco A., Velez-Delgado A., Menjivar R.E., Lima F., The S. (2021). Apolipoprotein E Promotes Immune Suppression in Pancreatic Cancer Through NF-κB-Mediated Production of CXCL1. Cancer Res..

[25] Murofushi Y., Villena J., Morie K., Kanmani P., Tohno M., Shimazu T., Aso H., Suda Y., Hashiguchi K., Saito T. (2015). The Toll-like Receptor Family Protein RP105/MD1 Complex Is Involved in the Immunoregulatory Effect of Exopolysaccharides from *Lactobacillus plantarum* N14. Mol. Immunol..

[26] Sun M., Liu W., Song Y., Tuo Y., Mu G., Ma F. (2021). The Effects of *Lactobacillus plantarum*-12 Crude Exopolysaccharides on the Cell Proliferation and Apoptosis of Human Colon Cancer (HT-29) Cells. Probiotics Antimicrob. Prot..

[27] Sahana T.G., Rekha P.D. (2019). A Bioactive Exopolysaccharide from Marine Bacteria *Alteromonas* sp. PRIM-28 and Its Role in Cell Proliferation and Wound Healing In Vitro. Int. J. Biol. Macromol..

[28] Alp G., Aslim B. (2010). Relationship Between the Resistance to Bile Salts and Low pH with Exopolysaccharide (EPS) Production of *Bifidobacterium* spp. Isolated from Infants Feces and Breast Milk. Anaerobe.

[29] Imran M.Y.M., Reehana N., Jayaraj K.A., Ahamed A.A.P., Dhanasekaran D., Thajuddin N., Alharbi N.S., Muralitharan G. (2016). Statistical Optimization of Exopolysaccharide Production by *Lactobacillus plantarum* NTMI05 and NTMI20. Int. J. Biol. Macromol..

[30] Jia K., Tao X., Liu Z., Zhan H., He W., Zhang Z., Zeng Z., Wei H. (2019). Characterization of Novel Exopolysaccharide of *Enterococcus faecium* WEFA23 from Infant and Demonstration of Its in Vitro Biological Properties. Int. J. Biol. Macromol..

[31] Blache P., van de Wetering M., Duluc I., Domon C., Berta P., Freund J.-N., Clevers H., Jay P. (2004). SOX9 Is an Intestine Crypt Transcription Factor, Is Regulated by the Wnt Pathway, and Represses the CDX2 and MUC2 Genes. J. Cell Biol..

[32] Qi Y., Wang D., Fang L., Liu X., Liu C., Zhao F., Wu D., Wang X., Wang J., Min W. (2022). Hypoglycemic Effect of Exopolysaccharide from *Lactiplantibacillus plantarum* JLAU103 on Streptozotocin and High-Fat Diet-Induced Type 2 Diabetic Mice. Foods.

[33] Li J., Qu C., Li F., Chen Y., Zheng J., Xiao Y., Jin Q., Jin G., Huang X., Jin D. (2020). Inonotus Obliquus Polysaccharide Ameliorates Azoxymethane/Dextran Sulfate Sodium-Induced Colitis-Associated Cancer in Mice via Activation of the NLRP3 Inflammasome. Front. Pharmacol..

[34] Tang J., Liu J., Yan Q., Gu Z., August A., Huang W., Jiang Z. (2021). Konjac Glucomannan Oligosaccharides Prevent Intestinal Inflammation Through SIGNR1-Mediated Regulation of Alternatively Activated Macrophages. Mol. Nutr. Food Res..

[35] Hutsko S.L., Meizlisch K., Wick M., Lilburn M.S. (2016). Early Intestinal Development and Mucin Transcription in the Young Poult with Probiotic and Mannan Oligosaccharide Prebiotic Supplementation. Poult. Sci..

[36] Wang K., Niu M., Yao D., Zhao J., Wu Y., Lu B., Zheng X. (2019). Physicochemical Characteristics and In Vitro and In Vivo Antioxidant Activity of a Cell-Bound Exopolysaccharide Produced by *Lactobacillus fermentum* S1. Int. J. Biol. Macromol..

[37] Zhang J., Xiao Y., Wang H., Zhang H., Chen W., Lu W. (2023). Lactic Acid Bacteria-Derived Exopolysaccharide: Formation, Immunomodulatory Ability, Health Effects, and Structure-Function Relationship. Microbiol. Res..

[38] Mao Y.-H., Song A.-X., Li L.-Q., Siu K.-C., Yao Z.-P., Wu J.-Y. (2020). Effects of Exopolysaccharide Fractions with Different Molecular Weights and Compositions on Fecal Microflora During In Vitro Fermentation. Int. J. Biol. Macromol..

[39] Li L.-Q., Song A.-X., Wong W.-T., Wu J.-Y. (2021). Isolation and Assessment of a Highly-Active Anti-Inflammatory Exopolysaccharide from Mycelial Fermentation of a Medicinal Fungus Cs-HK1. Int. J. Mol. Sci..

[40] Rubino S.J., Geddes K., Girardin S.E. (2012). Innate IL-17 and IL-22 Responses to Enteric Bacterial Pathogens. Trends Immunol..

[41] Kopan R., Ilagan M.X.G. (2009). The Canonical Notch Signaling Pathway: Unfolding the Activation Mechanism. Cell.

[42] Liu J., Xiao Q., Xiao J., Niu C., Li Y., Zhang X., Zhou Z., Shu G., Yin G. (2022). Wnt/β-Catenin Signalling: Function, Biological Mechanisms, and Therapeutic Opportunities. Signal Transduct. Target. Ther..

[43] Adesulu-Dahunsi A.T., Sanni A.I., Jeyaram K., Ojediran J.O., Ogunsakin A.O., Banwo K. (2018). Extracellular Polysaccharide from Weissella Confusa OF126: Production, Optimization, and Characterization. Int. J. Biol. Macromol..

